# Napor of Iodine in Mammary Tumors

**Published:** 1855-09

**Authors:** 


*Vapor of Iodine in Mammary Tumors.*—M. Eichmann relates the following case: A healthy woman, aged 47, none of whose relatives had ever had cancer, received a slight blow on the breast. This occasioned a hard and mobile tumor. After an absence of the catamenia for 17 months, the tumor became painful; smaller glandular enlargements appeared on the breast and extended to the axilla; lancinating pains were felt; the skin became adherent to the tumor, and the sebaceous follicles were distended with blackish matter. The patient refusing to submit to extirpation, M. Eichmann applied to the tumor a bag filled with tow, and containing also iodine, which was retained *in situ* by means of adhesive plaster. The iodine was renewed fortnightly. After wearing the bag for a month, the patient was greatly improved, and after the lapse of seventeen weeks the mammary enlargement had completely disappeared. —*Gazette des Hopitaux*, 19th December, 1854.

